# Association of Angiotensin-Converting Enzyme Genotype, Insertion/Deletion Polymorphism and Saphenous Vein Graft Atherosclerosis in Iranian Patients

**DOI:** 10.5935/1678-9741.20150069

**Published:** 2015

**Authors:** Neda Zeinali, Mohammad Hashemi, Mohsen Mirmohammadsadeghi, Hamid Mirmohammadsadeghi, Nahid Eskandari, Ali Mohammad Sabzghabaee

**Affiliations:** 1 School of Pharmacy and Pharmaceutical Sciences, Isfahan University of Medical Sciences, Isfahan, Iran.; 2 Department of Cardiology, School of Medicine, Isfahan University of Medical Sciences, Isfahan, Iran.; 3 Department of Cardiothoracic Surgery, School of Medicine, Isfahan University of Medical Sciences, Isfahan, Iran.; 4 Department of Pharmaceutical Biotechnology, School of Pharmacy, Isfahan University of Medical Sciences, Isfahan, Iran.; 5 Department of Immunology, School of Medicine, Isfahan University of Medical Sciences, Isfahan, Iran.; 6 Isfahan Clinical Toxicology Research Center, Isfahan University of Medical Sciences, Isfahan, Iran.

**Keywords:** Cardiopulmonary Bypass, Cardiovascular Surgical Procedures, Genetics

## Abstract

**OBJECTIVE:**

The aim of this study was to evaluate possible interactions among Angiotensin-I converting enzyme genotype, insertion/deletion polymorphism and atherosclerosis of vein grafts in Iranian patients, and characterize their clinical and demographic profile.

**METHODS:**

In this cross-sectional study, patients who underwent coronary artery bypass graft surgery more than five years ago, were included for angiographic analysis. Atherosclerosis was determined by quantitative angiography and adjusted Gensini score. The gene angiotensin converting enzyme I/D polymorphism was detected by polymerase chain reaction.

**RESULTS:**

A total of 102 patients participated in this study. Eighty-four patients were male. The frequency distribution of DD, ID and II polymorphism were 23.6%, 62.7% and 13.7% respectively. There were no differences among genotypic groups in age, sex, number of risk factors, number of vein grafts and months since bypass surgery. According to adjusted Gensini score [0.18±0.12 (II) *vs.* 0.11±0.09 (ID) and 0.1±0.09 (DD) *P*=0.021] the II genotype was associated with severity of vein graft atherosclerosis.

**CONCLUSION:**

Although there are conflicting results about gene angiotensin converting enzyme I/D polymorphism and the degree of venous bypass graft degeneration, this study suggests an association between ACE genotype II and atherosclerosis of saphenous vein grafts, however, large samples considering clinical, demographic and ethnic profile are necessary to confirm these results.

**Table:** 

**Abbreviations, acronyms & symbols**	
ACE	= Angiotensin-converting enzyme
CABG	= Coronary artery bypass graft
PCR	= Polymerase chain reaction

## INTRODUCTION

Coronary artery bypass graft surgery (CABG) still remains the standard care for patients with multivessel coronary artery disease comparing to percutaneous coronary intervention (PCI). This maybe due to its lower rates of major adverse cardiac and cerebrovascular events in short-term and long-term periods^[1-5]^.

A majority of patients receive saphenous vein grafting (SVG) to most vessels, with the exception of the left anterior descending coronary artery^[6]^. Large diameter and wall characteristics, being plentiful, long and easy harvest has made SVG the most commonly used conduit, but its longevity, as compared with arterial graft, is not satisfying. SVG patency is 95% at 1 week, 84% at 1 year, 80% at 3 years, 69% at 6 years, and 61% at 10 years after operation^[7]^. Thrombosis, intimal hyperplasia and atherosclerosis account for graft failure in early (less than 1 month), subacute (one to 12 months) and late (more than 12 months) post CABG periods^[8-11]^.

During past decades, many studies have shown genetic risk factors are as important as conventional risk factors in the development and progression of atherosclerosis^[12,13]^. Clinical assessment of the relationship between preoperative use of angiotensin-converting enzyme (ACE) inhibitors and clinical outcomes after CABG is discussed elsewhere^[14]^. One of the most significant genetic risk factors is the ACE gene insertion/deletion (I/D) polymorphism^[15]^. ACE encoding gene is located on chromosome 17 (17q23) and the presence (insertion) or absence (deletion) of a 287-bp alu repeat sequence in intron 16 of the gene results in ACE (I/D) polymorphism. This polymorphism accounts for 47% of variations in ACE levels^[16]^.

ACE is a key regulator in the renin angiotensin system (RAS) which converts Angiotensin I into Angiotensin II (Ang II)^[17]^. Ang II, the atherogenic component of the RAS, develops atheroma. Ang II increases vascular permeability and induces expression of inflammatory mediators such as cytokines, chemokines and adhesion molecules^[18]^. It also stimulates proliferation and migration of vascular smooth muscle cells and extracellular matrix deposition^[19]^. Ang II plays a role in oxidative stress by activating nicotinamide dinucleotide phosphate (NADPH) oxidase to produce reactive oxygen species (ROS)^[20,21]^.

To our best of knowledge, the association of ACE (I/D) polymorphism and venous bypass graft degeneration in long-term periods has not been studied in Iranian patients, so the aim of this study was to evaluate possible interactions between ACE (I/D) polymorphism and atherosclerosis of vein grafts in Iranian patients, and characterize their clinical and demographic profile.

## METHODS

The research protocol of this cross-sectional study was approved by the institutional board of research ethics for human studies at the Isfahan University of Medical Sciences (Research project number # 389305).

### Study population

Study population consisted of 102 patients who have undergone CABG more than 5 years ago and were referred to Sina Hospital and Noor University Hospital (located in Isfahan, Iran) for coronary angiography due to coronary heart disease (CHD) symptoms from December 2010 to October 2011.Medical histories of all patients were obtained and CHD conventional risk factors were evaluated according to these criteria: high blood pressure (systolic blood pressure >140 mmHg and diastolic blood pressure >90 mmHg or taking antihypertensive agents), hypercholesterolemia (total cholestero >240 mg/dL or taking lipid lowering agents), diabetes mellitus (fasting blood glucose >110 mg/dL or taking anti hyperglycemic agents), cigarette smoking (daily habit) and positive family history (men <55 years old and women <65 years old in first degree of relatives).

### Vein graft angiography

Cardiac catheterization and vein graft angiography was performed using Judkins technique^[22]^ and percutaneos femoral artery approach. Vein graft bed was divided into three segments: proximal, middle and distal. The severity of venous bypass graft atherosclerosis was quantified according to Gensini score. Gensini score was defined by grading of reduction in vessel lumen diameter. Narrowing of 25%, 50%, 75%, 90%, 99% and 100% were equivalent to Gensini score of 1, 2, 4, 8, 16 and 32 respectively^[23]^. The Gensini score of three segments of each vein graft were added and the mean of the Gensini score of total vein grafts in each patient was obtained to reflect the degree of atherosclerosis. For illustrating the rate of bypass degeneration, the mean of Gensini score was divided by the months after CABG (adjusted Gensini score: The mean of Gensini score/months after surgery).

### Determination of ACE genotypes

Genomic DNAs were extracted from whole blood leucocytes using DNA isolation kit (High Pure PCR Template Preparation kit, Roche Diagnostics GmbH, Germany). Extracted DNAs were stored at -20°C for future polymerase chain reaction (PCR). Two PCRs with four primers were carried out for detection of ACE intron 16 I/D polymorphism. The first PCR primers were 5'-CTG GAG ACC ACT CCC ATC CTT TCT-3' (forward primer) and 5'GAT GTG GCC ATC ACA TTC GTC AGA T-3' (reverse primer). Final volume of PCR reaction mixture was 25µl that contained 12.5 pmol of each primer, 2.8 mM MgCl2, 0.8 mM of each dNTp, 2.5 µl of 10X High Fidelity PCR Buffer (Fermentase) and 1 U of Taq polymerase (High Fidelity PCR Enzyme Mix, Fermentase).

Amplification was performed in DNA Thermal Cycler (Analytic Jena) with initial denaturation step at 94°C for 5 min followed by 30 cycles consisting of denaturation at 94°C for 1 min, annealing at 58°C for 1 min and extension at 72°C for 2 min, followed by final extension step at 72°C for 5 min. PCR products were separated on 1% agarose gel and DNA was visualized by ethidium bromide staining on UV transillumination imaging system. DNA fragment sizes were 190 bp for the D allele and 490 bp for the I allele.

To avoid DD mistyping, second PCR was carried out on DD genotype samples^[24]^. Second PCR was performed with an specific insertion primer pairs included 5'-TGG GAC CAC AGC GCC CGC CAC TAC-3' as a forward primer and 5'-TCG CCA GCC CTC CCA TGC CCA TAA-3' as a reverse primer in 25 µl reaction mixture volume. Second PCR steps consisted of initial denaturation at 94°C for 1 min followed by 30 cycles of denaturation at 94°C for 30 s, annealing at 67°C for 45 s and extension at 72°C for 2 min followed by final extension at 72°C for 5 min. Positive control samples (samples showed II genotype in first PCR) were used to confirm the reliability of genotyping. Presence of I allele resulted in PCR product of 335 bp.

### Statistical analysis

Statistical analysis was carried out using SPSS version 20.0 and data were expressed as the mean±SD or percentage. Chi-square test, Fisher's exact test or Kruskal-Wallis test (where appropriate) was used for non-parametric variables and independent t-student test or one-way ANOVA test was used for analyzing the numerical data. *P*<0.05 was considered statistically significant. If statistically significant *P* value obtained by ANOVA, Bonferroni's post-hoc test was performed for pair wise comparison.

## RESULTS

### Demographic characteristics of the study population

Demographic characteristics and clinical features of the study population are shown in Table 1. The mean of months since CABG was 128.7±38.36. The proportion of occluded, diseased and free of atherosclerosis vein grafts were 42.12%, 26% and 31.88% respectively.

**Table 1:** Demographic characteristics and clinical features of the studied patients with vein graft atherosclerosis.

Parameter	N (%) / Mean±SD
n	102
Sex (Female/Male)	18 (17.6)/84 (82.4)
Age (year)	64.89±8.54
Body Mass Index (kg/m^2^)	26.52±3.68
Hypertension (positive)	62 (60.8)
Hypercholesterolemia (positive)	95 (93.1)
Diabetes mellitus (positive)	30 (29.4)
Cigarette smoking (positive)	17 (16.7)
Family history (positive)	68 (66.7)
Total Coronary Heart Disease risk factors	3 (1-5)*
Total number of vein grafts	254
Left anterior descending	12
Left circumflex	5
Diagonal	54
Optus margin	95
Right coronary artery	81
Ramous	7
Occluded vein grafts	107
Diseased vein grafts	66
Free of atherosclerosis	81
Number of vein grafts per patient	3 (1-5)*
Months elapsed from Coronary Artery Bypass Graft surgery	128.7±38.36
Vein graft Gensini score**	13.99±11.41
Adjusted vein graft Gensini score†	0.11±0.1

* Median (range)

** Vein graft Gensini score: the mean of Gensini score of total grafts in each patient

† Adjusted vein graft Gensini score: vein graft Gensini score was divided by months elapsed from Coronary Artery Bypass Graft surgery

### Genotype and allele frequencies

The genotype and allele frequencies are listed in Table 2. Observed genotype and allele frequencies of ACE I/D polymorphism were in Hardy-Weinberg equilibrium in the study population.

**Table 2:** Genotype and allele frequencies of ACE I/D polymorphism in studied patients with vein graft atherosclerosis.

ACE I/D polymorphism	Genotype	N (%)	Allele	N (Absolute frequency)
	II	14 (13.7)	D	112 (0.55)
	ID	64 (62.7)	I	92 (0.45)
	DD	24 (23.6)		

ACE=Angiotensin Converting Enzyme

### Relationship between ACE I/D polymorphism and progression of atheromatous plaque in vein graft

Relationship between ACE I/D polymorphism and severity of vein graft atherosclerosis and comparison of the number of occluded, diseased and free of atherosclerosis vein grafts, number of vein grafts, months after CABG and number of CHD risk factors according to genotype are reported in Table 3. According to Gensini score, ACE I/D polymorphism was associated with progression and development of atherosclerosis in venous bypass grafts (*P*=0.02). Bonferroni's post hoc- test showed patients who were homozygous for I allele had higher adjusted Gensini score and rate of atherosclerosis (Table 4). Although there was no statistical difference in the number of diseased vein grafts per genotype, Gensini score showed bypass degeneration was more severe in II genotype. Without regarding adjusted Gensini score, ACE II genotype resulted in vein graft failure in earlier postoperative period (statistically marginal significant difference in months after CABG, *P*=0.06).

**Table 3:** Effect of ACE I/D polymorphism on progression of atherosclerosis in vein grafts of the studied patients.

ACE I/D polymorphism	II	ID	DD	*P*
Adjusted Gensini score, Mean (± SD)	0.18±0.12	0.11+0.09	0.1+0.09	0.02*
Number of occluded vein grafts, Median (Range)	1 (0-4)	1 (0-3)	1 (0-4)	0.64**
Number of diseased vein grafts, Median (Range)	0 (0-2)	1 (0-2)	0.5 (0-3)	0.84**
Number of free of atherosclerosis vein grafts, Median (Range)	0 (0-2)	1 (0-3)	1 (0-4)	0.27**
Number of vein grafts, Median (Range)	2 (1-4)	2 (1-5)	3 (1-5)	0.41**
Months post CABG, Mean (± SD)	1 12.07±32.46	135.02±38.33	121.04±38.67	0.06*
Number of CHD risk factors, Median (Range)	2 (1-4)	3 (0-5)	2.5 (1-4)	0.58**

ACE=angiotensin converting enzyme, CABG=coronary artery bypass graft surgery

* One-wayAnova test,

** Kruskal-Wallis test

**Table 4:** Comparison of angiographic severity of coronary artery disease in different ACE genotypes.

ACE genotype	Adjusted Gensini score	*P*
II *vs.* ID	0.18±0.12 *vs.* 0.11±0.09	0.029*
II *vs.* DD	0.18±0.12 *vs.* 0.1±0.09	0.032*
ID *vs.* DD	0.11±0.09 *vs.* 0.1±0.09	1*

ACE=angiotensin converting enzyme, independent t-student test

## DISCUSSION

The results of our study show that ACE II genotype could be considered a risk factor for long-term graft failure after CABG. ACE II may have some roles in the progression of atheromatous plaque of vein grafts in earlier postoperative periods as compared with two other genotypic groups. Few studies about the influence of ACE I/D polymorphism on venous bypass graft atherosclerosis are available. Studies carried out in Turkey and Germany demonstrated different results^[25-27]^. Ortlepp et al.^[25]^ reported ACE I/D polymorphism was not associated with venous bypass degeneration in the long term in their 101 studied patients. Dayi et al.^[26]^ indicated DD genotype influenced vein graft occlusion in late postoperative period in their 87 consecutively selected patients. On the other hand, our study shows no statistical difference among ACE genotypic groups and the number of occluded vein grafts (*P*=0.64, Kruskal-Wallis test). In a study done by Völzke et al.^[27]^ on 247 patients, they demonstrated ACE DD genotype increased the rate of mortality and cardiovascular morbidity in the midterm after CABG.

Although ACE DD genotype was associated with the increasing risk of cardiovascular disease in many studies, some studies reported other type of genotypes as risk factors. Zee et al.^[28]^ showed I allele was risk factor for essential hypertension. Ismail et al.^[29]^ found significantly higher frequency of ACE II genotype in hypertensive patients aged 20-40 years. In Northern Indian population I allele was associated with essential hypertension^[30]^. ACE ID genotype was responsible for peripheral vascular disease in Western Turkish patients^[31]^.

The results of the study of ACE I/D polymorphism and cardiovascular disease in Iranian population seem conflicting. The presence of D allele exacerbated the risk of early onset coronary artery disease in west population of Iran^[32]^. Shafiee et al.^[33]^ showed different results. They reported no association between ACE DD genotype and the risk of coronary artery disease. They collected study population from patients referred to Shahid Rajaei Cardiovascular Medical and Research Center, Tehran, Iran^[33]^. Despite the adverse effect of ACE DD genotype on hypertension in type 2 diabetic population reported by Nakhjavani et al.^[34]^, Nikzamir et al.^[35]^ found no relation between ACE I/D polymorphism and the presence of metabolic syndrome in patients with type 2 diabetes.

In our study the frequency distribution of II, ID and DD genotype are 13.7%, 62.7% and 23.6% respectively. These values are similar to the results found by Nikzamir et al.^[35]^ (16.5%, 58.2% and 25.3%), but are different from the data reported by Vaisi-Raygani et al.^[32]^ (17.3%, 40% and 42.7%), Shafiee et al.^[33]^ (16.22%, 32.24% and 51.51%) and Nakhjavani et al.^[34]^ (27.5%, 50% and 22.5%). Frequency distribution of ACE I/D polymorphism observed in various parts of Iran is different and it may be responsible for the discrepancies in the reported results. It seems that different ACE I/D polymorphism interactions with cardiovascular disease could be attributed to the various ethnicities studied in previously mentioned Iranian studies.

We also think that the heterogeneity of our sample considering clinical profile, pharmacological treatment and lifestyle, as well as the surgical technique for myocardial revascularization, could influence the follow-up as well as its relation with the genetic profile. Lack of serum ACE activity measurement and also the small sample size are main limitations of this study.

## CONCLUSION

Although there are conflicting results about ACE I/D polymorphism and the degree of venous bypass graft degeneration, this study suggests an association between ACE genotype II and atherosclerosis of saphenous vein grafts, however, large samples considering clinical, demographic and ethnic profile are necessary to confirm these results.

**Table:** 

**Authors' roles & responsibilities**		
NZ	Collected the data and helped in data analysis; final approval of the manuscript	
MH	Contributed in designing and conducting the study; final approval of the manuscript	
MM	Contributed in designing and conducting the study; final approval of the manuscript	
HM	Rechecked the statistical analysis and revised the manuscript; final approval of the manuscript	
NE	Rechecked the statistical analysis and revised the manuscript; final approval of the manuscript	
AMS	Proposed the idea; managed the research project; rechecked the statistical analysis; prepared the manuscript and final approval of the manuscript	

## References

[1] Marui A, Kimura T, Nishiwaki N, Mitsudo K, Komiya T, Hanyu M, CREDO-Kyoto PCI/CABG Registry Cohort-2 Investigators (2014). Comparison of five-year outcomes of coronary artery bypass
grafting versus percutaneous coronary intervention in patients with left
ventricular ejection fractions &le; 50% versus &gt;50% (from the
CREDO-Kyoto PCI/CABG Registry Cohort-2). Am J Cardiol.

[2] Serruys PW, Morice MC, Kappetein AP, Colombo A, Holmes DR, Mack MJ, SYNTAX Investigators (2009). Percutaneous coronary intervention versus coronary-artery bypass
grafting for severe coronary artery disease. N Engl J Med.

[3] Daemen J, Boersma E, Flather M, Booth J, Stables R, Rodriguez A (2008). Long-term safety and efficacy of percutaneous coronary
intervention with stenting and coronary artery bypass surgery for
multivessel coronary artery disease: a meta-analysis with 5-year
pa-tient-level data from the ARTS, ERACI-II, MASS-II, and SoS
trials. Circulation.

[4] Hlatky MA, Boothroyd DB, Bravata DM, Boersma E, Booth J, Brooks MM (2009). Coronary artery bypass surgery compared with percutaneous
coronary interventions for multivessel disease: a collaborative analysis of
individual patient data from ten randomised trials. Lancet.

[5] Sá MPBO, Ferraz PE, Escobar RR, Nunes EO, Soares AMMN, Sá FBCA (2013). Five-year outcomes following PCI with DES versus CABG for
unprotected LM coronary lesions: meta-analysis and meta-regression of 2914
patients. Rev Bras Cir Cardiovasc.

[6] Sen O, Gonca S, Solakoglu S, Dalcik H, Dalcik C, Ozkara A (2013). Comparison of conventional and no-touch techniques in harvesting
saphenous vein for coronary artery bypass grafting in view of endothelial
damage. Heart Surg Forum.

[7] Sabik 3rd JF (2011). Understanding saphenous vein graft patency. Circulation.

[8] Harskamp RE, Lopes RD, Baisden CE, de Winter RJ, Alexander JH (2013). Saphenous vein graft failure after coronary artery bypass
surgery: pathophysiology, management, and future directions. Ann Surg.

[9] Shukla N, Jeremy JY (2012). Pathophysiology of saphenous vein graft failure: a brief overview
of interventions. Curr Opin Pharmacol.

[10] Parang P, Arora R (2009). Coronary vein graft disease: pathogenesis and
prevention. Can J Cardiol.

[11] Belczak CEQ, Tyszka AL, Godoy JMP, Ramos RN, Belczak SQ, Caffaro RA (2009). Clinical complications of limb undergone harvesting of great
saphenous vein for coronary artery bypass grafting using bridge
technique. Rev Bras Cir Cardiovasc.

[12] Roy H, Bhardwaj S, Yla-Herttuala S (2009). Molecular genetics of atherosclerosis. Hum Genet.

[13] Arnett DK, Baird AE, Barkley RA, Basson CT, Boerwinkle E, Ganesh SK, American Heart Association Council on Epidemiology and
Prevention, American Heart Association Stroke Council, Functional Genomics and Translational Biology Interdisciplinary
Working Group (2007). Relevance of genetics and genomics for prevention and treatment
of cardiovascular disease: a scientific statement from the American Heart
Association Council on Epidemiology and Prevention, the Stroke Council, and
the Functional Genomics and Translational Biology Interdisciplinary Working
Group. Circulation.

[14] Radaelli G, Bodanese LC, Guaragna JCVC, Borges AP, Goldani MA, Petracco JB (2011). The use of inhibitors of angiotensin-converting enzyme and its
relation to events in the postoperative period of CABG. Rev Bras Cir Cardiovasc.

[15] Zintzaras E, Raman G, Kitsios G, Lau J (2008). Angiotensin-converting enzyme insertion/deletion gene polymorphic
variant as a marker of coronary artery disease: a
meta-analysis. Arch Intern Med.

[16] Urhan Küçük M, Sucu N, Sahan Firat S, Aytaçoglu BN, Vezir Ö, Bozali C (2014). Role of ACE I/D gene polymorphisms on the effect of ramipril in
inflammatory response and myocardial injury in patients undergoing coronary
artery bypass grafts. Eur J Clin Pharmacol.

[17] Teranishi J, Yamamoto R, Nagasawa Y, Shoji T, Iwatani H, Okada N (2015). ACE insertion/deletion polymorphism (rs1799752) modifies the
renoprotective effect of renin-angiotensin system blockade in patients with
IgA nephropathy. J Renin Angiotensin Aldosterone Syst.

[18] Pacurari M, Kafoury R, Tchounwou PB, Ndebele K (2014). The Renin-Angiotensin-aldosterone system in vascular inflammation
and remodeling. Int J Inflam.

[19] Fang H, Chen W, Gao Y, Shen Y, Luo M (2014). Molecular mechanisms associated with Angiotensin-converting
enzyme-inhibitory peptide activity on vascular extracellular matrix
remodeling. Cardiology.

[20] Zablocki D, Sadoshima J (2013). Angiotensin II and oxidative stress in the failing
heart. Antioxid Redox Signal.

[21] Dikalov SI, Nazarewicz RR, Bikineyeva A, Hilenski L, Lassègue B, Griendling KK (2014). Nox2-induced production of mitochondrial superoxide in
angiotensin II-mediated endothelial oxidative stress and
hypertension. Antioxid Redox Signal.

[22] Oldroyd KG, Phadke KV, Phillips R, Carson PH, Clarke M, Davis JA (1989). Cardiac catheterisation by the Judkins technique as an outpatient
procedure. BMJ.

[23] Gensini GG (1983). A more meaningful scoring system for determining the severity of
coronary heart disease. Am J Cardiol.

[24] Shanmugam V, Sell KW, Saha BK (1993). Mistyping ACE heterozygotes. PCR Methods Appl.

[25] Ortlepp JR, Janssens U, Bleckmann F, Lauscher J, Merkelbach-Bruse S, Hanrath P (2001). A chymase gene variant is associated with atherosclerosis in
venous coronary artery bypass grafts. Coron Artery Dis.

[26] Dayi SU, Tartan Z, Terzi S, Kasikcioglu H, Uyarel H, Orhan G (2005). Influence of angiotensin converting enzyme insertion/deletion
polymorphism on long-term total graft occlusion after coronary artery bypass
surgery. Heart Surg Forum.

[27] Völzke H, Engel J, Kleine V, Schwahn C, Dahm JB, Eckel L (2002). Angiotensin I-converting enzyme insertion/deletion polymorphism
and cardiac mortality and morbidity after coronary artery bypass graft
surgery. Chest.

[28] Zee RY, Lou YK, Griffiths LR, Morris BJ (1992). Association of a polymorphism of the angiotensin I-converting
enzyme gene with essential hypertension. Biochem Biophys Res Commun.

[29] Ismail M, Akhtar N, Nasir M, Firasat S, Ayub Q, Khaliq S (2004). Association between the angiotensin-converting enzyme gene
insertion/deletion polymorphism and essential hypertension in young
Pakistani patients. J Biochem Mol Biol.

[30] Srivastava K, Sundriyal R, Meena PC, Bhatia J, Narang R, Saluja D (2012). Association of angiotensin converting enzyme (insertion/deletion)
gene polymorphism with essential hypertension in northern Indian
subjects. Genet Test Mol Biomarkers.

[31] Basar Y, Salmayenli N, Aksoy M, Seçkin S, Aydin M, Ozkök E (2007). ACE gene polymorphism in peripheral vascular
disease. Horm Metab Res.

[32] Vaisi-Raygani A, Ghaneialvar H, Rahimi Z, Nomani H, Saidi M, Bahrehmand F (2010). The angiotensin converting enzyme D allele is an independent risk
factor for early onset coronary artery disease. Clin Biochem.

[33] Shafiee SM, Firoozrai M, Salimi S, Zand H, Hesabi B, Mohebbi A (2010). Angiotensin converting enzyme DD genotype not associated with
increased risk of coronary artery disease in the Iranian
population. Pathophysiology.

[34] Nakhjavani M, Esfahanian F, Jahanshahi A, Esteghamati A, Nikzamir AR, Rashidi A (2007). The relationship between the insertion/deletion polymorphism of
the ACE gene and hypertension in Iranian patients with type 2
diabetes. Nephrol Dial Transplant.

[35] Nikzamir A, Nakhjavani M, Golmohamadi T, Dibai L (2008). Association of angiotensin-converting enzyme gene
insertion/deletion polymorphism with metabolic syndrome in Iranians with
type 2 diabetes mellitus. Arch Iran Med.

